# Risk factors for infections in patients with multiple myeloma: a systematic review and meta-analysis

**DOI:** 10.3389/fonc.2026.1726340

**Published:** 2026-03-12

**Authors:** Qiulian He, Yi He, Zhihui Wang, Xiaorong Zhao

**Affiliations:** 1 Department of Hematology, Beijing Anzhen Nanchong Hospital of Capital Medical University & Nanchong Central Hospital, Nanchong, China; 2 Department of Orthopedics, People’s Hospital of Nanbu County, Nanchong, China

**Keywords:** infection, meta-analysis, multiple myeloma, risk factors, systematic review

## Abstract

**Background:**

Multiple myeloma (MM) is a malignant clonal disease originating from plasma cells. Patients with MM are prone to infections due to factors such as impaired immune function, bone marrow suppression, and anticancer therapy. A systematic review and meta-analysis of risk factors for infections in patients with MM provides evidence-based guidance for clinical risk assessment and intervention.

**Methods:**

Search databases such as PubMed, Web of Science, Embase, Cochrane Library, from the establishment of the database to July 20, 2025, to include observational studies evaluating risk factors for infection in patients with MM. Use the NOS tool for literature quality assessment and perform meta-analysis using Stata 15.0 to combine odds ratios (OR) and 95% confidence intervals (CI).

**Results:**

This study included 15 articles (1 case-control study and 14 cohort studies), involving 4,717 participants. The results of this meta-analysis suggest that age > 65 [OR = 1.72, 95% CI (1.18, 2.48)], male [OR = 1.70, 95% CI (1.11, 2.62)], smoking [OR = 2.97, 95% CI (1.94, 4.57)], International Staging System III [OR = 2.22, 95% CI (1.81, 2.73)], diabetes [OR = 2.67, 95% CI (1.74, 4.09)], immunomodulatory drugs [OR = 3.40, 95% CI (2.29, 5.07)], hemoglobin <10 g/dL[OR = 2.28, 95% CI (1.65, 3.16)], creatinine ≥ 2 mg/dL[OR = 2.80, 95% CI (2.07, 3.79)] was significantly associated with infection in patients with multiple myeloma.

**Conclusion:**

This systematic review and meta-analysis indicate that age >65 years, male gender, smoking, ISS stage III, diabetes, use of immunosuppressive agents, hemoglobin <10 g/dL, and creatinine ≥2 mg/dL are all significant risk factors for infection in MM patients.

**Systematic review registration:**

https://www.crd.york.ac.uk/prospero/, identifier CRD420251065706

## Background

Multiple myeloma (MM) is a type of hematological malignancy caused by the malignant clonal proliferation of plasma cells (1). It is characterized by abnormal proliferation of monoclonal plasma cells in the bone marrow, accompanied by the secretion of large amounts of immunoglobulins or their fragments (M proteins), leading to a series of clinical manifestations such as bone destruction, anemia, hypercalcemia, renal dysfunction, and immune dysfunction (2, 3). MM is the third most common hematological malignancy after non-Hodgkin lymphoma and acute myeloid leukemia, accounting for approximately 1% of all malignant tumors and about 10% of hematological malignancies (4, 5). With the global aging population and improved diagnostic capabilities, the incidence of MM has been increasing annually. According to the Global Cancer Statistics (GLOBOCAN 2022), the incidence rate of MM is approximately 1.8 cases per 100,000 population annually, with higher rates in developed countries in Europe and America, reaching 4–6 cases per 100,000 (6). The incidence rate of MM in China is lower than in Europe and America, but it has also been gradually increasing in recent years (7). MM primarily affects the elderly population, with a median age of onset of approximately 65–70 years, and the incidence rate is slightly higher in males than in females (8). Although the survival period of MM patients has significantly increased in recent years with the application of treatment methods such as proteasome inhibitors (bortezomib), immunomodulators (lenalidomide, pomalidomide), monoclonal antibodies (such as daratumumab), and autologous hematopoietic stem cell transplantation, the median overall survival (OS) has reached more than 10 years (9, 10). However, treatment-related complications have become increasingly prominent, with infection being one of the leading causes of death in MM patients. It has been reported that MM patients face a high risk of infection throughout the course of the disease, accounting for approximately 15% to 25% of deaths, with the risk being most pronounced during the initial diagnosis and early stages of treatment (11, 12).

MM patients experience a variety of infections, with bacterial infections being the most common, particularly respiratory and bloodstream infections caused by Gram-negative and Gram-positive bacteria (13). Common pathogens include Streptococcus pneumoniae, Escherichia coli, Klebsiella pneumoniae, and Staphylococcus aureus. Viral infections are more prominent in patients receiving immunomodulators or monoclonal antibody therapy, such as varicella-zoster virus (VZV) and respiratory syncytial virus (RSV) infections. Fungal infections are common in patients receiving long-term corticosteroid or broad-spectrum antibiotic therapy, particularly Aspergillus and Candida infections (14, 15). Once these infections occur, they often progress rapidly, are difficult to control, and have a poor prognosis.

Infection is a critical issue in the management of prognosis for MM patients. As treatment modalities continue to evolve, the survival period of MM patients has been extended, and infection-related mortality and disability issues have become increasingly prominent (16). Although various measures have been implemented clinically, such as prophylactic antibiotics, antiviral drugs, and vaccination, the lack of unified high-quality evidence-based data has left prevention and intervention strategies incomplete (17). Through systematic reviews and meta-analyses, existing evidence is integrated and quantitatively analyzed to identify the key risk factors for infections in MM patients, providing evidence-based guidance for clinical risk stratification and the development of preventive measures.

## Methods

This systematic evaluation and meta-analysis will strictly follow the PRISMA (Preferred Reporting Items for Systematic Reviews and Meta-Analyses) guidelines (18). And it is registered in Prospero with registration number CRD420251065706.

### Inclusion and exclusion criteria

Inclusion criteria: Study participants must be patients with a confirmed diagnosis of multiple myeloma, with no restrictions on gender, age, race, or region; study type is limited to observational studies, including cohort studies and case-control studies; the study must report the incidence of infection in MM patients and its associated risk factors, and be able to directly provide or calculate the odds ratio and its 95% confidence interval based on raw data.

Exclusion criteria include the following aspects: studies where the study subjects were not clearly diagnosed with multiple myeloma, or studies that combined analysis with other hematological malignancies and could not extract relevant data separately, will be excluded; reviews, case reports, conference abstracts, animal experiments, *in vitro* experiments, editorials, and commentaries will not be included; studies that did not involve infection risk factors or did not provide relevant effect values and could not be calculated based on raw data will also be excluded; Additionally, studies with incomplete data, data that cannot be extracted or converted into statistical indicators, will not be included; if the same study population is published in multiple literature sources, only the study with the most complete, latest, or largest sample size will be included.

### Literature retrieval

Two researchers (HQL and HY) conducted independent systematic searches of the following databases: PubMed, Web of Science, Embase, and the Cochrane Library. The search period for all databases was from the inception of each database to July 20, 2025. The search strategy combined Medical Subject Headings (MeSH) with free-text terms, consisting of three main components: “Multiple Myeloma,” “Infection,” and “Risk factor.” The specific search strategy is detailed in **Supplementary Material Table S1**. The search strategy was adjusted appropriately for each database based on its characteristics. To further ensure the comprehensiveness of the literature, this study also manually searched the reference lists of included studies to supplement any potentially overlooked relevant research. In cases of disagreement during the search and screening process, a third researcher was involved to mediate and resolve the issue.

### Study selection

During the literature screening process, two researchers (HQL and HY) independently used EndNote 21 software to initially screen the literature obtained from the search, first through the titles and abstracts, and then to exclude literature that clearly did not meet the inclusion criteria. Subsequently, the remaining literature was reviewed by reading the full text in its entirety to further determine whether it met the inclusion and exclusion criteria. In case of disagreement between the two researchers during the screening process, it would be resolved through discussion and negotiation; if the negotiation still failed to reach a consensus, a third researcher would be invited to adjudicate to ensure the objectivity and consistency of the screening process.

### Data extractions

This study was conducted by two researchers (HQL and HY) who independently extracted relevant data from the eligible literature using an Excel sheet based on the inclusion criteria. The extraction included the basic information of the study (first author, year of publication, country and study design), the basic characteristics of the study population (sample size, number of infections, type of infections, gender, and mean age), the statistical model used in the regression analysis, and diagnosis of cognitive impairment. In the process of data extraction, if two investigators disagreed on the data, it would be resolved through negotiation, and if no agreement could be reached, a third investigator would adjudicate to ensure the accuracy and consistency of data extraction.

### Quality evaluation

The types of studies included in this study will be assessed using different quality assessment tools: for case-control and cohort studies, the NOS (Newcastle-Ottawa Scale) (19) quality assessment tool will be used, which evaluates the intrinsic bias of the studies through three main domains (study selectivity, comparability, and assessment of outcomes), focusing on sample selection, the relationship between exposure and relationship between exposure and outcome, and control of confounders; these quality assessment tools ensure that the included studies have a high-quality evidence base.

### Statistical analysis

In this study, the risk ratio (OR) and the corresponding 95% confidence interval (CI) of each included study were combined using Stata 15 software. First, for each study, we extracted the corresponding effect size OR and its 95% confidence interval. To combine these ORs, we pooled them using a random effects model, which can account for heterogeneity between studies, variability in effect sizes across studies. ORs and 95% CIs were calculated for each study and combined into an overall effect size. Heterogeneity of the model was assessed by the I² statistic; if the I² was greater than 50%, it was considered that there was a high degree of heterogeneity and that the sources of heterogeneity needed to be further explored (20). For high heterogeneity, we may conduct sensitivity analyses to identify potential factors that may affect the combined effect sizes. Asymmetry in the funnel plot indicates a higher likelihood of publication bias, which will be further evaluated using Egger’s test. P value < 0.05 suggests the presence of publication bias, while a P value > 0.05 suggests otherwise. If necessary, the trim-and-fill method will be used for further confirmation. The combined effect sizes will be reported as ORs and their 95% CIs to allow for interpretation of results and statistical inference.

## Results

### Literature screening results

**Figure 1:**
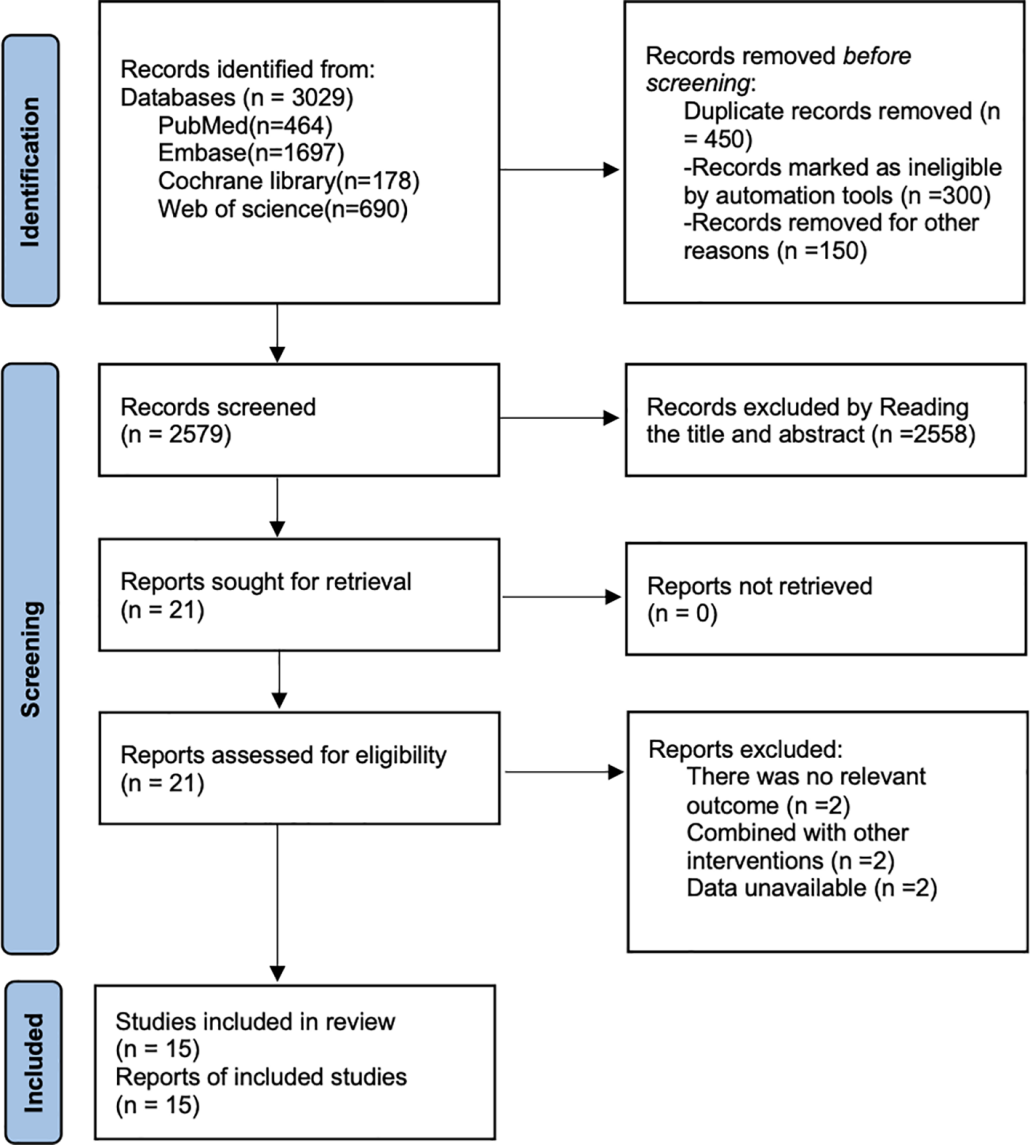
Literature search flow chart.

As shown in **Figure 1**, a total of 3,029 articles were retrieved from PubMed (n=464), Embase (n=1,697), Cochrane Library (n=178), and Web of Science (n=690). After removing 450 duplicate documents, 2,558 articles were excluded based on title and abstract review, and 6 articles were excluded after full-text review. Ultimately, 15 articles (21–35) were included in the analysis.

### Basic characteristics of the included literature

This study included 15 articles, including 1 case-control study (21) and 14 cohort studies (22–35), with a total of 4,717 participants. The studies were conducted across multiple geographic regions, including China (n = 7), the United States (n = 2), Uruguay (n = 2), and one study each from Korea, Australia, Poland, and Denmark. The total sample size ranged from 68 to 2557 participants, with infection events varying substantially across studies. Reported infection types included bacterial infections alone, fungal infections alone, viral infections alone, or combinations of bacterial, viral, and fungal infections, reflecting heterogeneity in outcome definitions. Most studies reported mixed infection categories, while several focused specifically on bacterial infections. Across studies, the mean age of participants ranged from 57 to 70 years, and the gender distribution was generally balanced. Logistic regression was the most used analytical method to estimate associations between risk factors and infection outcomes, whereas several studies employed Cox proportional hazards models. The specific baseline characteristics are shown in **Table 1**.

**Table 1:** Table of basic characteristics.

Study	Year	Country	Study design	Sample size	No of infection	Types of infection	Gender (M/F)	Mean age (years)	Regression model
Baneman (21)	2025	USA	Case-control	123	31	Fungal	65/58	62	logistic regression
Bove (23)	2022	Uruguay	Cohort Study	248	89	Bacteria+Viral	135/113	64	logistic regression
Bici (22)	2023	USA	Cohort Study	144	21	Bacteria	88/56	64	logistic regression
Huang (24)	2017	China	Cohort Study	222	26	Bacteria	158/84	64	Cox regression
Huang (25)	2021	China	Cohort Study	123	55	Bacteria+Viral	70/53	57	Cox regression
Jung (26)	2014	Korea	Cohort Study	155	28	Bacteria	78/77	61	logistic regression
Li (27)	2021	China	Cohort Study	85	47	Bacteria+Fungal	45/40	63.1	logistic regression
Lim (28)	2021	Australia	Cohort Study	148	45	Bacteria+Viral+Fungal	92/56	66	logistic regression
Lin (29)	2020	China	Cohort Study	161	41	Bacteria+Viral+Fungal	94/67	60	logistic regression
Mikulski (30)	2022	Poland	Cohort Study	174	54	Bacteria+Viral+Fungal	84/90	65	Cox regression
Pan (31)	2025	China	Cohort Study	230	86	Viral	127/103	65	logistic regression
Riva (32)	2024	Uruguay	Cohort Study	124	54	Bacteria	65/59	65	logistic regression
Sorrig (33)	2019	Denmark	Cohort Study	2557	1176	Bacteria+Viral+Fungal	1410/1147	70	Cox regression
Yang (34)	2024	China	Cohort Study	155	125	Bacteria+Viral+Fungal	88/67	63	logistic regression
Zhou (35)	2023	China	Cohort Study	68	43	Bacteria+Viral+Fungal	45/23	57.5	Cox regression

### Risk of bias results

This study used the NOS score for evaluation. Nine articles (21–23, 26, 27, 30, 31, 34, 35) scored 9 points, three articles (24, 28, 32) scored 8 points, and three articles (25, 29, 33) scored 7 points. All included studies scored above 7 points, indicating high-quality research. The specific evaluation results are shown in **Table 2**.

**Table 2:** NOS score results.

Case control										
Study		Is the case definition adequate?	Representativeness of the cases	Determination of control group	Definition of Controls	Comparability of cases and controls based on the design or analysis	Ascertainment of exposure	Same method of ascertainment for cases and controls	Non response	Total scores
Baneman2025 (21)		*	*	*	*	**	*	*	*	9
Cohort study										
Study	Representativeness of the exposed group		Selection of non-exposed groups	Determination of exposure factors	Identification of outcome indicators not yet to be observed at study entry	Comparability of exposed and unexposed groups considered in design and statistical analysis	design and statistical analysis	Adequacy of the study's evaluation of the outcome	Adequacy of follow-up in exposed and unexposed groups	Total scores
Bove2022 (23)	*		*	*	*	**	*	*	*	9
Bici2023 (22)	*		*	*	*	**	*	*	*	9
Huang2017 (24)	*		*	/	*	**	*	*	*	8
Huang2021 (25)	*		*	/	*	*	*	*	*	7
Jung2014 (26)	*		*	*	*	**	*	*	*	9
Li2021 (27)	*		*	*	*	**	*	*	*	9
Lim2021 (28)	*		*	/	*	**	*	*	*	8
Lin2020 (29)	*		*	/	*	*	*	*	*	7
Mikulski2022 (30)	*		*	*	*	**	*	*	*	9
Pan2025 (31)	*		*	*	*	**	*	*	*	9
Riva2024 (32)	*		*	/	*	**	*	*	*	8
Sorrig2019 (33)	*		*	/	*	*	*	*	*	7
Yang2024 (34)	*		*	*	*	**	*	*	*	9
Zhou2023 (35)	*		*	*	*	**	*	*	*	9

* one scores, ** two scores.

### Meta analysis results

Eight potential risk factors for infection in patients with MM were synthesized. Overall, between-study heterogeneity was low across analyses (I² ranging from 0% to 19.2%), and therefore fixed-effects models were applied.

**Figure 2:**
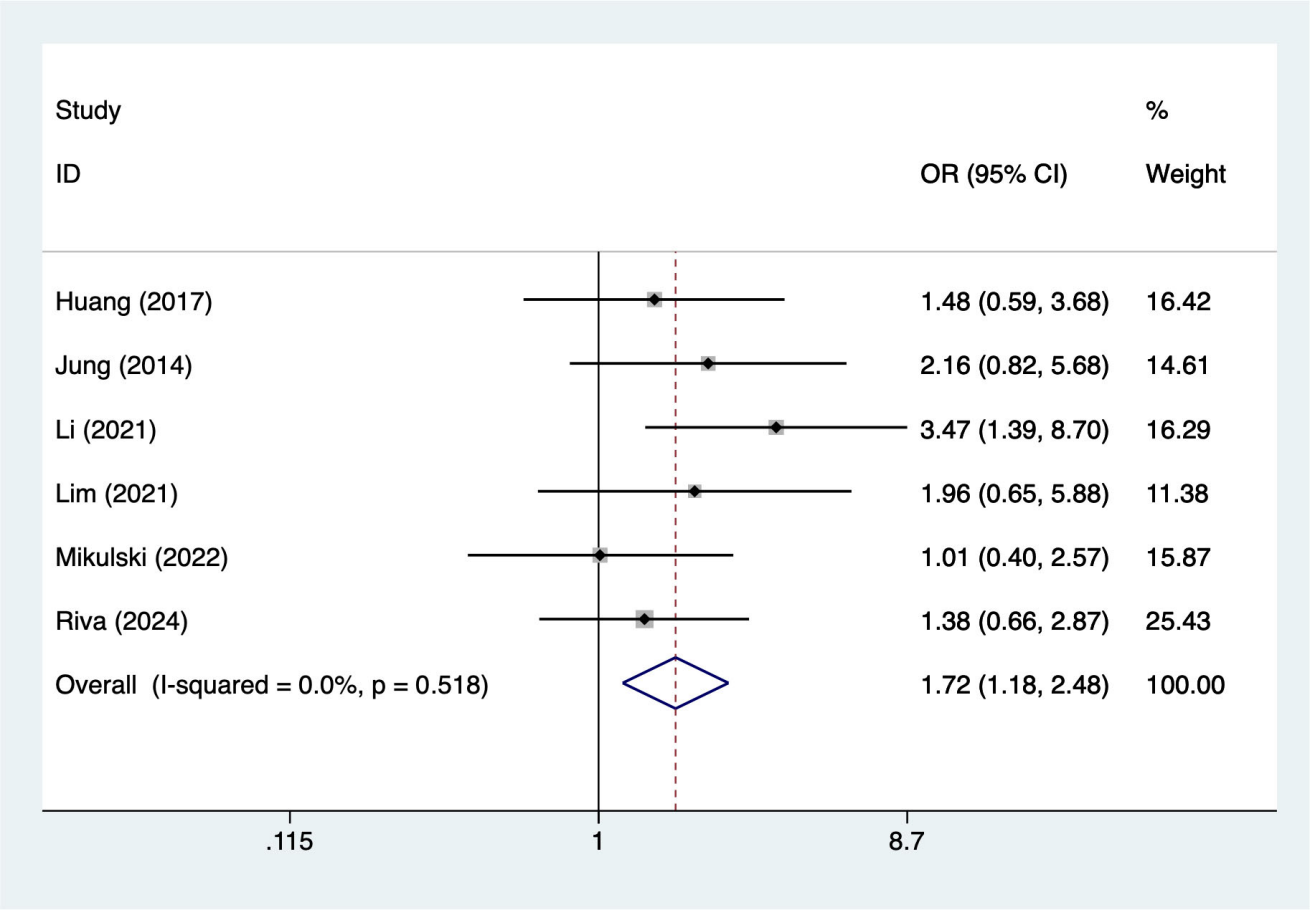
The forest plot of the meta-analysis indicates that age > 65 is a risk factor for infection in multiple myeloma.

**Figure 3:**
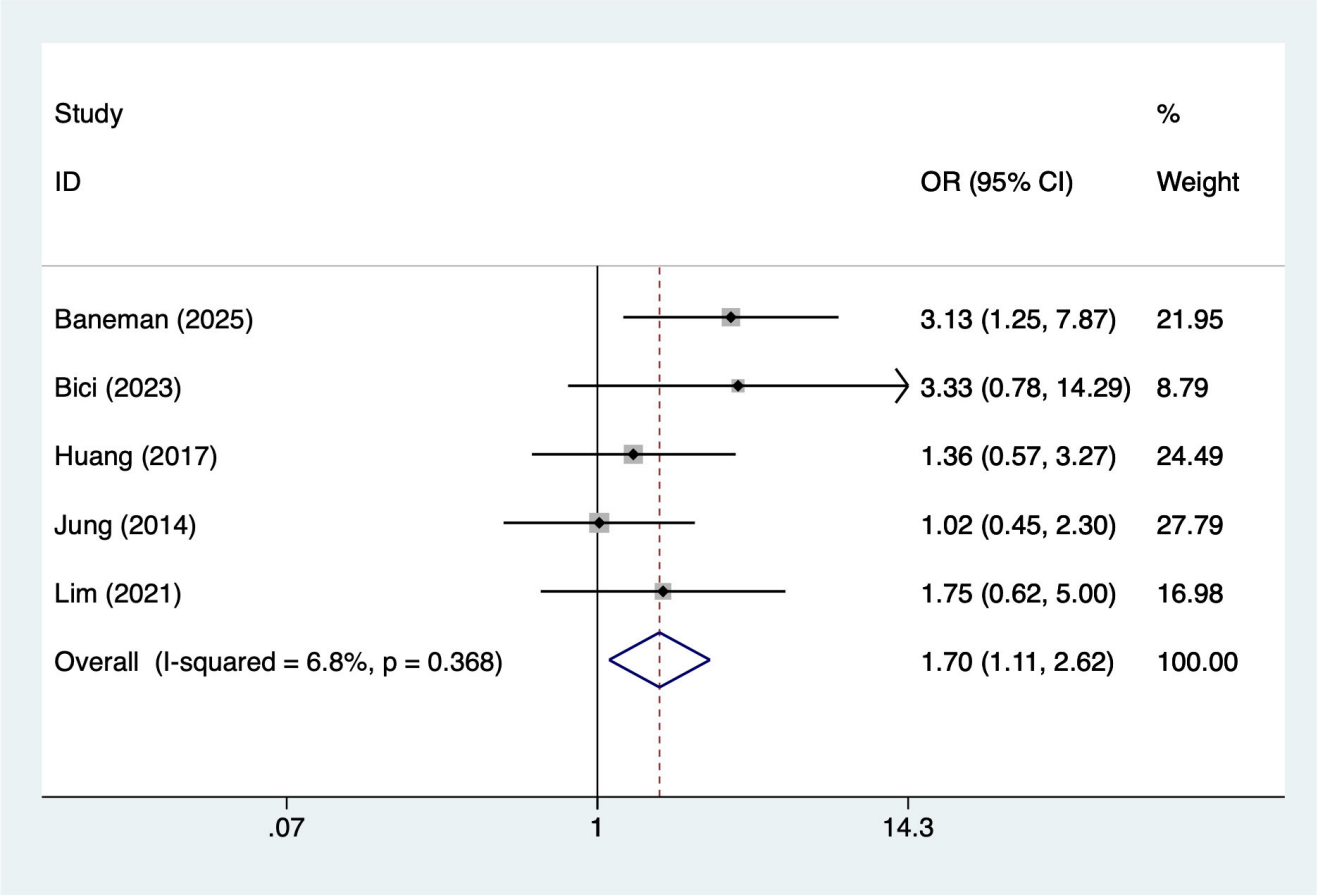
The forest plot of the meta-analysis indicates that male is a risk factor for infection in multiple myeloma.

**Figure 4:**
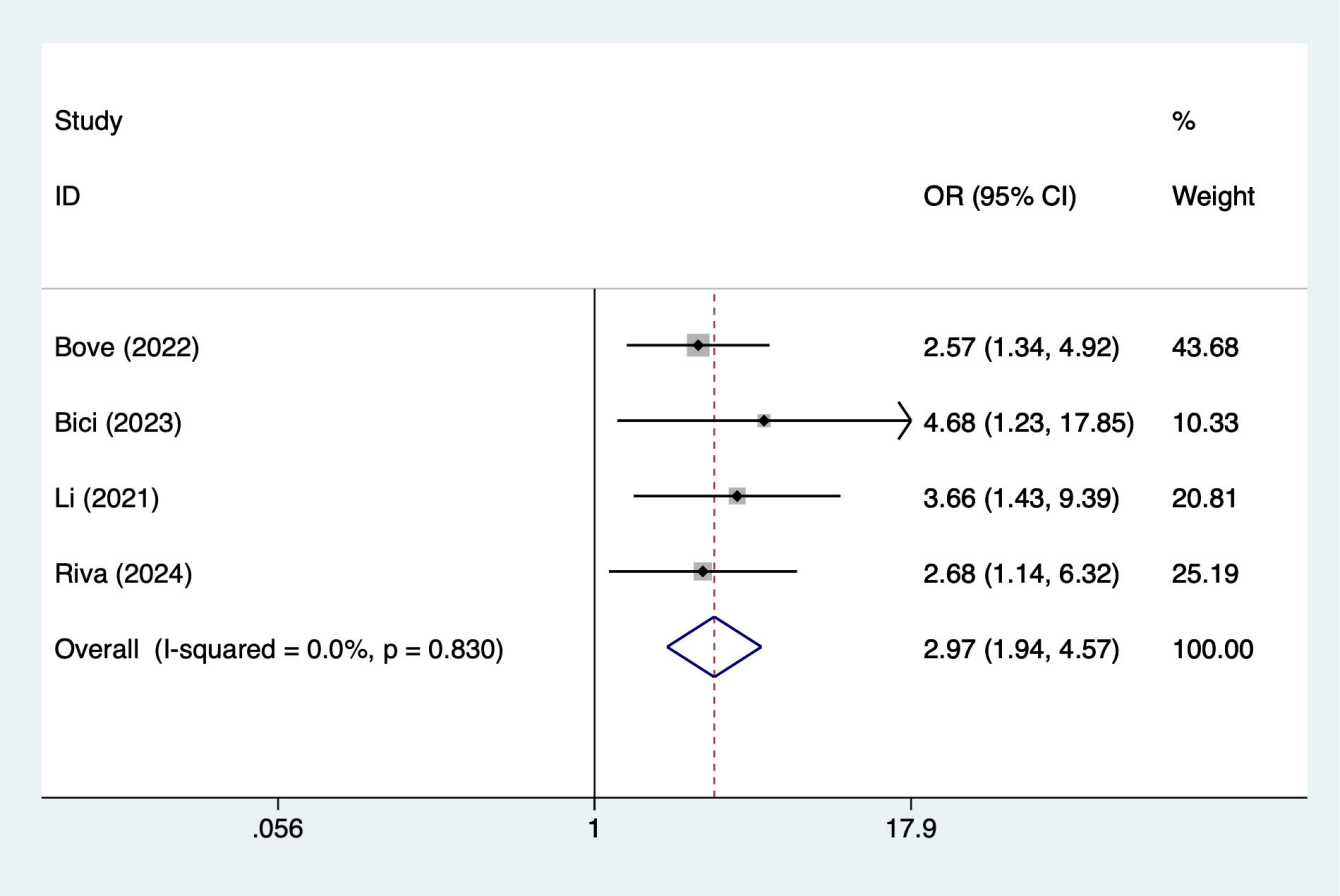
The forest plot of the meta-analysis indicates that smoking is a risk factor for infection in multiple myeloma.

Older age (>65 years) was associated with an increased risk of infection (OR = 1.72, 95% CI 1.18–2.48; **Figure 2**). Similarly, male sex showed a significant association (OR = 1.70, 95% CI 1.11–2.62; **Figure 3**). Lifestyle-related risk was also observed, as smoking nearly tripled the infection risk (OR = 2.97, 95% CI 1.94–4.57; **Figure 4**).

**Figure 5:**
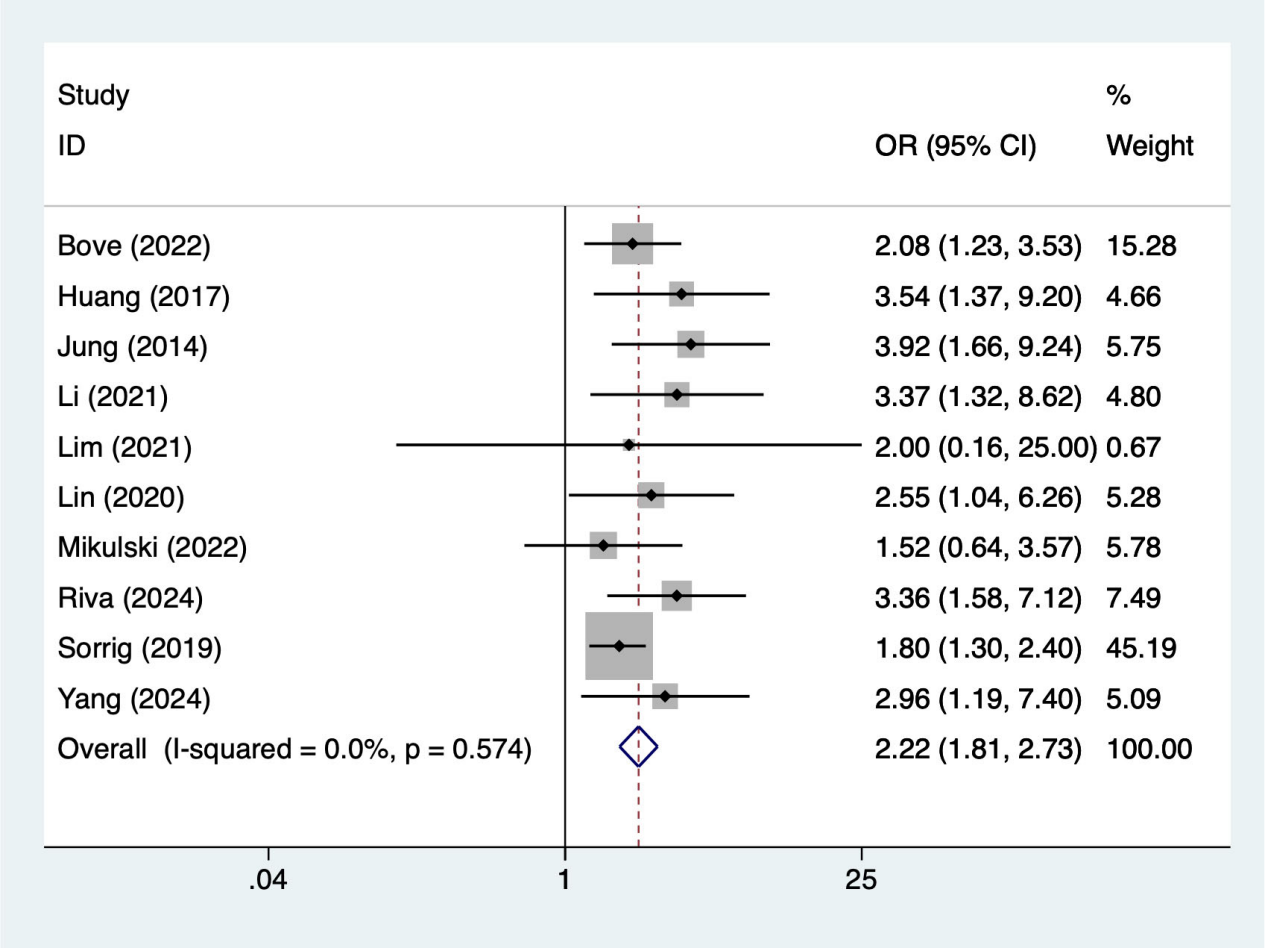
The forest plot of the meta-analysis indicates that ISS stage III is a risk factor for infection in multiple myeloma.

**Figure 6:**
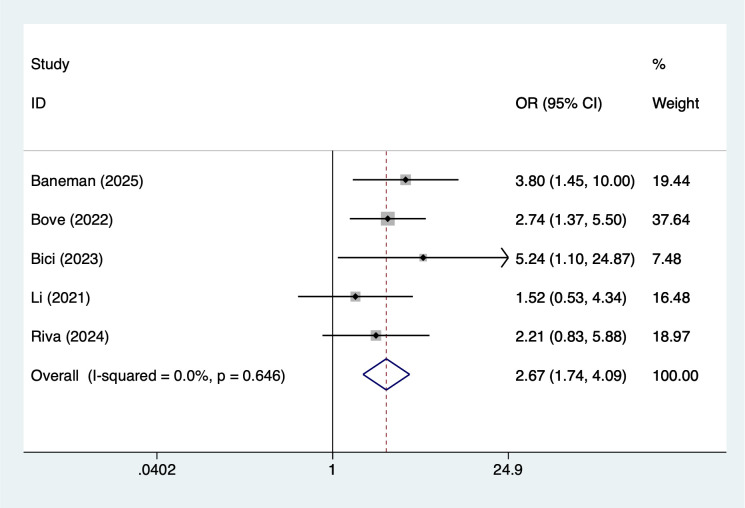
The forest plot of the meta-analysis indicates that diabetes is a risk factor for infection in multiple myeloma.

Disease severity and comorbidity factors demonstrated consistent and comparatively stronger associations. Patients with International Staging System stage III had more than double the risk of infection (OR = 2.22, 95% CI 1.81–2.73; **Figure 5**), and diabetes was similarly associated with increased susceptibility (OR = 2.67, 95% CI 1.74–4.09; **Figure 6**).

**Figure 7:**
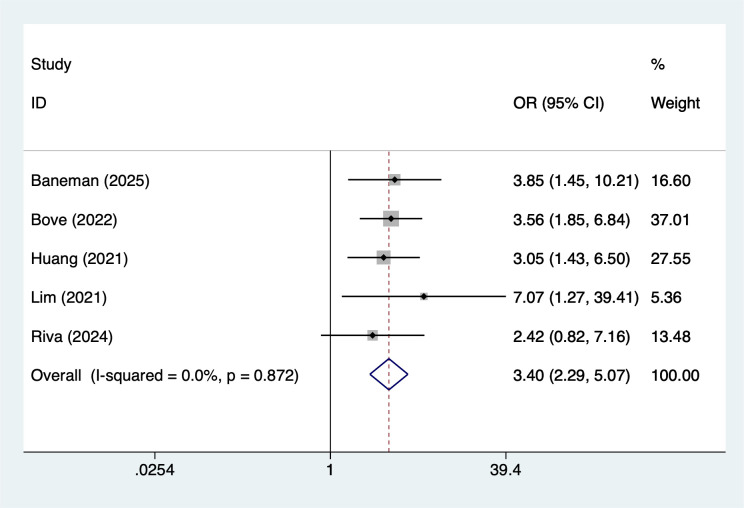
The forest plot of the meta-analysis indicates that immunosuppressive agents is a risk factor for infection in multiple myeloma.

**Figure 8:**
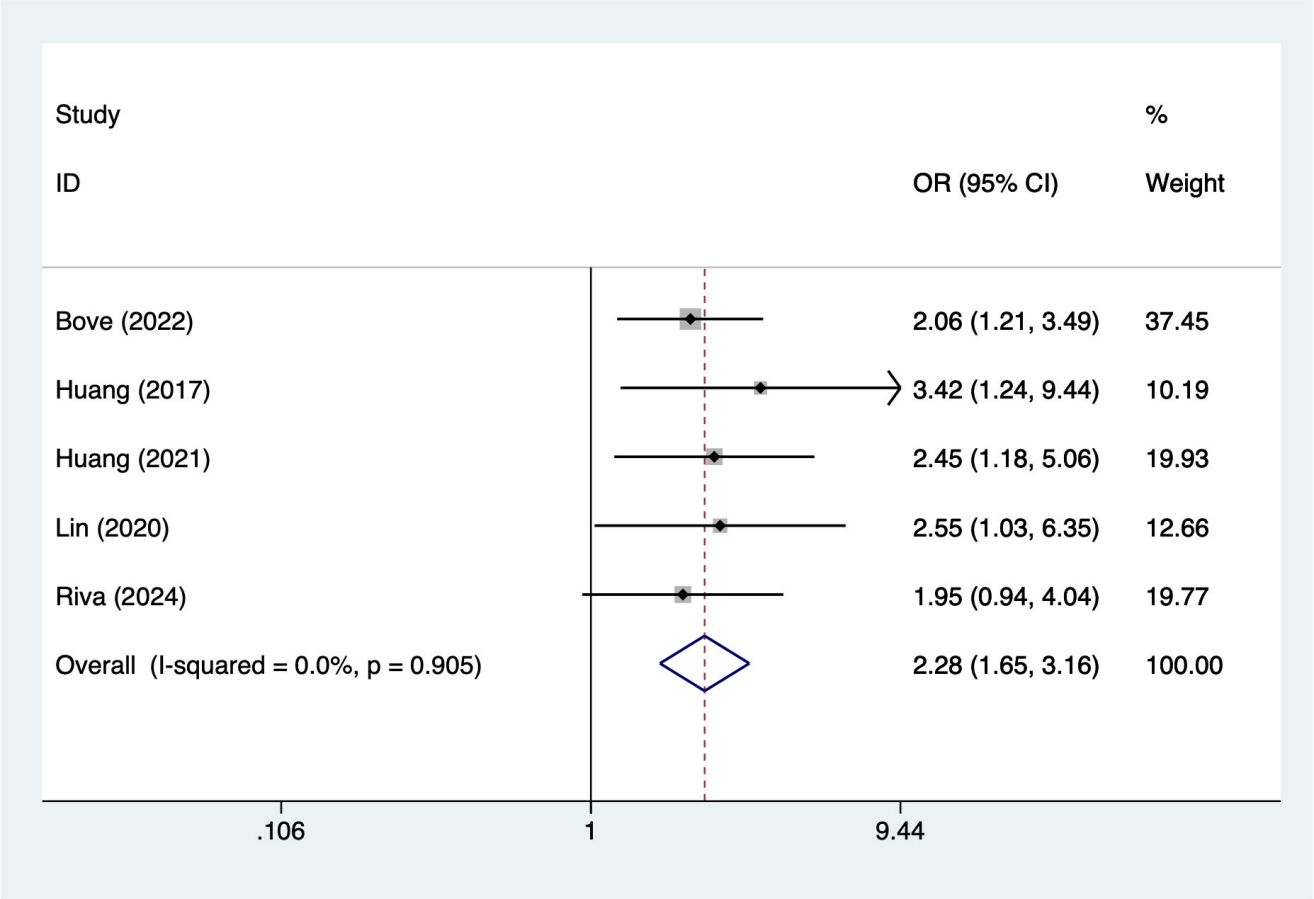
The forest plot of the meta-analysis indicates that hemoglobin <10 g/dL is a risk factor for infection in multiple myeloma.

**Figure 9:**
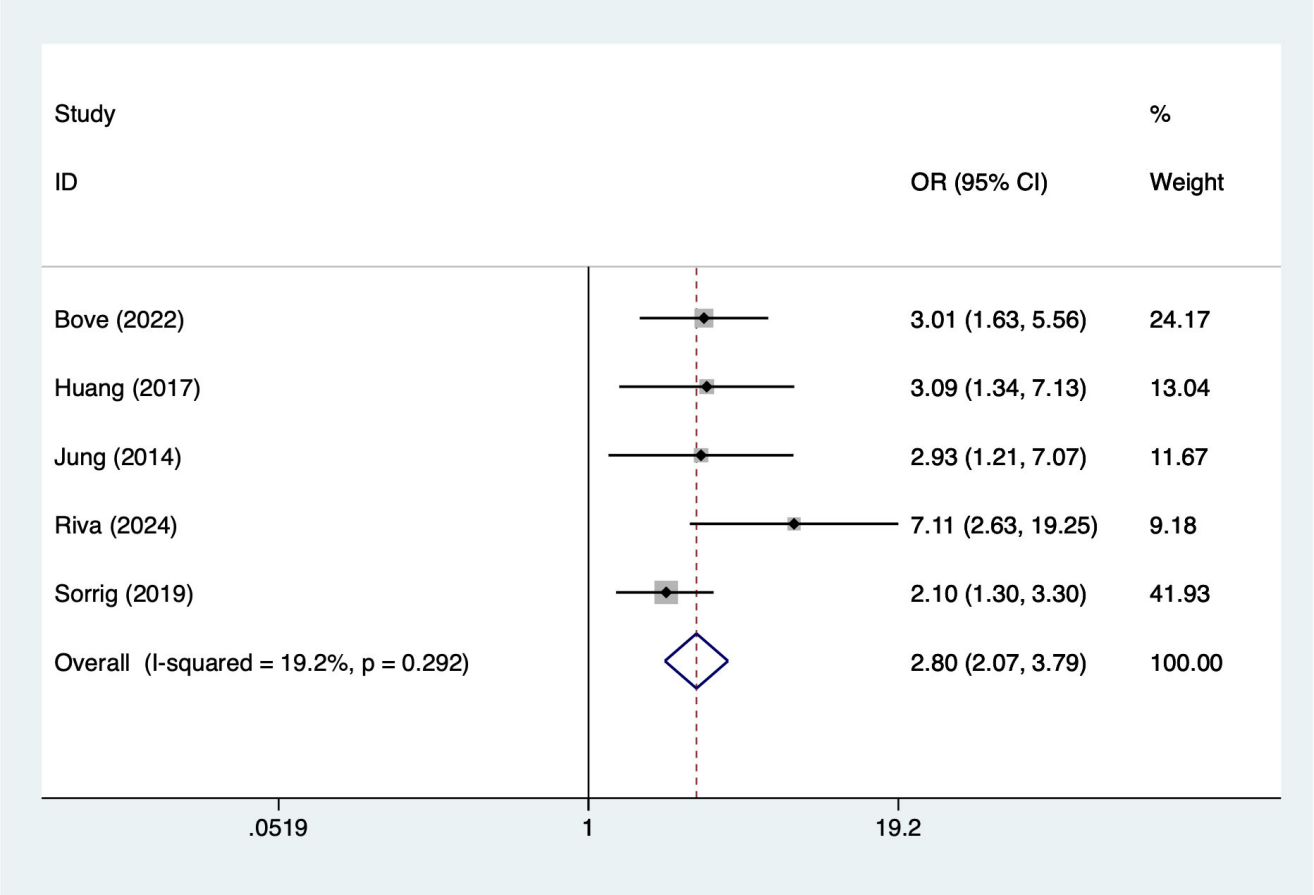
The forest plot of the meta-analysis indicates that creatinine ≥2 mg/dL is a risk factor for infection in multiple myeloma.

Treatment-related and laboratory indicators were also significantly associated with infection outcomes. Use of immunomodulatory drugs showed the strongest observed association (OR = 3.40, 95% CI 2.29–5.07; **Figure 7**). Hemoglobin <10 g/dL (OR = 2.28, 95% CI 1.65–3.16; **Figure 8**) and creatinine ≥2 mg/dL (OR = 2.80, 95% CI 2.07–3.79; **Figure 9**) were both linked to elevated infection risk.

Taken together, these findings indicate that infection risk in MM patients is multifactorial, involving demographic characteristics, disease burden, comorbid conditions, treatment exposure, and laboratory abnormalities, with particularly strong associations observed for smoking and immunomodulatory drug use.

### Publication bias

This study used funnel plots and the Egger test to assess publication bias. The funnel plot results (**Supplementary Materials Figures S1**-**S8**) indicated symmetrical distribution on both sides of the funnel plot, suggesting a low likelihood of publication bias. The Egger test results indicated no significant association with age >65 (P = 0.654), male gender (P = 0.262), smoking (P = 0.081), International Staging System III (P = 0.464), diabetes (P = 0.698), immunomodulatory drugs (P = 0.052), hemoglobin < 10 g/dL (P = 0.104), and creatinine ≥ 2 mg/dL (P = 0.076).

## Discussion

### Key findings

This systematic review and meta-analysis integrated 15 studies, involving a total of 4,717 patients with multiple myeloma, to systematically assess the relationship between various clinical and treatment-related factors and the risk of infection. The analysis results showed that age >65 years, male gender, smoking, ISS stage III, diabetes, use of immunomodulators, hemoglobin <10 g/dL, and creatinine ≥2 mg/dL were all significantly associated with an increased risk of infection. These findings provide important evidence for clinicians to identify high-risk populations and develop individualized prevention and control measures in the management of MM patients.

### Association between age > 65 and infection in MM patients

This study shows that age over 65 significantly increases the risk of infection in MM patients (OR = 1.72). This result is consistent with previous studies. Elderly patients often have impaired immune function, including thymic atrophy, reduced T-cell activity, decreased B-cell production, and impaired immunoglobulin response (36). Additionally, elderly patients often have multiple underlying conditions, such as diabetes, chronic obstructive pulmonary disease, and cardiovascular disease, which further weaken the body’s ability to resist infection (37). In terms of treatment, elderly patients have impaired organ reserve function, making them less tolerant of intensive chemotherapy and hematopoietic stem cell transplantation, often requiring adjustments to treatment regimens (38). This may lead to poor disease control, thereby indirectly increasing the risk of infection. Furthermore, some studies (39, 40) have indicated that elderly MM patients have lower vaccine response rates, suggesting that traditional preventive measures may have limited efficacy. However, it should be noted that the present meta-analysis did not differentiate infection types (bacterial, viral, or fungal) and did not include data on plasma immunoglobulin levels. Therefore, our findings do not directly support specific prophylactic interventions. In clinical practice, beyond routine infection control measures, additional strategies such as intravenous immunoglobulin (IVIG) or individualized antimicrobial prophylaxis may be considered based on patient-specific risk assessment and existing guidelines, rather than being inferred from the current analysis. Future studies with detailed microbiological characterization and immunological profiling are needed to clarify these preventive approaches.

### Association between male and infection in MM patients

This study found that male MM patients have a higher risk of infection than females (OR = 1.70). Gender differences may be related to the interaction between the endocrine and immune systems (41). Estrogen in women has certain immune-protective effects, enhancing humoral and cellular immune functions, while androgens may have immunosuppressive effects (42). Additionally, men have higher rates of lifestyle factors such as smoking and alcohol consumption, which are risk factors for infection and may partially explain the gender differences. It is worth noting that some studies suggest that gender differences are more pronounced in certain specific infections (43). For example, male patients have a higher incidence of pneumonia and urinary tract infections, while women do not have a significantly increased risk of fungal infections. This suggests that clinical prevention and control efforts should prioritize screening and prevention of respiratory and urinary tract infections in male patients.

### Association between smoking and infection in MM patients

Smoking is significantly associated with an increased risk of infection in MM patients (OR = 2.97), with the highest risk magnitude observed in this analysis. Smoking damages the respiratory and immune systems through multiple mechanisms (44). First, smoking directly impairs the ciliary function of respiratory epithelial cells, weakening the mechanical barrier function and making it easier for pathogens to invade. Second, harmful components in tobacco can suppress macrophage function and neutrophil chemotaxis, reducing the body’s ability to clear bacteria and viruses. Additionally, smoking is highly associated with chronic bronchitis and chronic obstructive pulmonary disease (COPD), which are independent risk factors for infection themselves (45). In MM patients, the negative effects of smoking on the immune system may compound the immune deficiency effects of the disease itself, significantly increasing the risk of infection. Therefore, smoking cessation interventions should be an integral part of comprehensive patient management, particularly in patients receiving strong immunosuppressive therapy.

### Association between international staging system III and infection in MM patients

The results of this study indicate that patients with ISS stage III have a significantly higher risk of infection compared to those with stage I or II (OR = 2.22). ISS staging is primarily based on serum β2-microglobulin and albumin levels, which are important indicators reflecting the disease burden and prognosis of MM (46). Stage III patients typically indicate a high tumor burden, severe immunosuppression, and poor overall health, all of which are high-risk factors for infection. Additionally, patients in the advanced stages of the disease often require more intensive chemotherapy and combination therapy regimens, such as proteasome inhibitors combined with immunomodulators or monoclonal antibodies, which further exacerbate immune system damage (47, 48). Clinically, patients with ISS stage III should be considered a high-risk group for infection. In addition to routine antimicrobial prophylaxis measures, stricter monitoring should be implemented, including regular hematological examinations, pathogen screening, and the early use of prophylactic antimicrobial agents when necessary.

### Association between diabetes and infection in MM patients

Diabetes is an independent risk factor for infection in MM patients (OR = 2.67). Diabetic patients have impaired immune system function, manifested as reduced neutrophil phagocytic function, weakened chemotaxis, and abnormal humoral immunity. Additionally, a hyperglycemic environment promotes the growth and reproduction of bacteria and fungi, particularly Candida and Aspergillus, significantly increasing the incidence of opportunistic infections (49). In MM treatment, corticosteroids are commonly used medications, and corticosteroids exacerbate blood glucose fluctuations, further increasing the infection risk in diabetic patients. Previous studies have shown that patients with diabetes and MM not only have a higher infection rate but also experience faster disease progression and poorer treatment outcomes once an infection occurs (50). Therefore, clinical management should prioritize glycemic control and individualized medication selection for patients with diabetes and MM, and prophylactic antifungal and antiviral treatments should be considered when necessary.

### Association between immunomodulatory drugs and infection in MM patients

Immune modulating agents (IMiDs) such as lenalidomide and pomalidomide are widely used in the treatment of MM(MM). This study demonstrated a significant association between their use and the risk of infection (OR = 3.40). IMiDs exert their antitumor effects by enhancing T-cell and NK-cell function, but they can also cause bone marrow suppression, cytopenia, and immune dysfunction, thereby increasing the risk of infection (51). This risk is particularly elevated when IMiDs are used in combination with glucocorticoids or proteasome inhibitors. For example, the combination of IMiDs with bortezomib has been shown to increase the risk of varicella-zoster virus (VZV) infection. Therefore, when using IMiDs for treatment, routine VZV prophylaxis (acyclovir) should be administered, and blood cell counts should be closely monitored.

### Association between hemoglobin <10g/dL and infection in MM patients

Anemia (Hb <10 g/dL) is extremely common in MM patients, and this study found it to be significantly associated with infection risk (OR = 2.28). Anemia reflects suppressed bone marrow hematopoietic function and disease progression, and it can also lead to tissue hypoxia and impaired immune cell function, thereby weakening the body’s ability to resist pathogens. Additionally, anemia often indicates poor nutritional status in patients, and malnutrition itself is a risk factor for infection (52). Previous study has shown that MM patients with severe anemia have a significantly higher probability of developing pulmonary infections and sepsis compared to non-anemic patients (53). Therefore, improving anemia not only helps alleviate symptoms but may also indirectly reduce the risk of infection.

### Association between creatinine ≥ 2 mg/dL and infection in MM patients

Renal dysfunction is one of the common complications of MM. The results of this study indicate that patients with serum creatinine ≥2 mg/dL have a significantly increased risk of infection (OR = 2.80). Renal dysfunction not only affects drug metabolism and clearance but also leads to impaired immune function in a uremic environment, such as reduced complement activity and impaired lymphocyte function (54). Additionally, renal dysfunction is often accompanied by electrolyte imbalances and acid-base disturbances, which can create conditions conducive to the onset and exacerbation of infections. Furthermore, patients with renal dysfunction may require adjustments to medication doses or even discontinuation of certain drugs during treatment, which may impact disease control and indirectly increase the risk of infection (55). Therefore, in MM patients, renal function should be closely monitored, and renal protective strategies should be implemented in advance for high-risk patients. Antimicrobial prophylaxis should be considered when necessary.

### Clinical implications for infection prevention

From a clinical perspective, the findings support a risk-adapted prevention strategy rather than a uniform prophylactic approach. Baseline patient-related factors should guide early interventions such as optimization of comorbidity management, smoking cessation counseling, nutritional support, and individualized vaccination planning. Meanwhile, disease- and treatment-related risks warrant dynamic surveillance during therapy, including hematologic monitoring, timely pathogen screening, and consideration of guideline-supported antimicrobial or antiviral prophylaxis in selected high-risk populations.

Overall, infection vulnerability in MM arises from the interplay between host characteristics and therapeutic exposure. Recognizing these overarching patterns may facilitate more precise targeting of preventive measures and improve supportive care planning. Future research should aim to refine this framework through incorporation of infection subtype data, immunological biomarkers, and longitudinal risk prediction models to enable more personalized infection prevention strategies.

### Context within existing literature

Overall, our findings are broadly consistent with prior studies identifying advanced age, comorbidity burden, and disease severity as major contributors to infection risk in MM, supporting the external validity of these associations. The observed relationship between immunomodulatory therapy and infection also aligns with clinical trial safety data reporting increased susceptibility to viral reactivation and cytopenia-related complications. However, not all findings are uniformly supported in the literature. For instance, sex differences in infection risk remain inconsistent across cohorts, and the impact of anemia or renal dysfunction varies in magnitude between studies. These discrepancies may reflect heterogeneity in study populations, infection definitions, treatment regimens, and adjustment for confounding variables. Additionally, limited reporting of infection subtypes and immune parameters across studies restricts direct comparison and mechanistic interpretation.

### Strengths and limitations

The strengths of this study lie in its systematic and comprehensive approach, which encompasses multiple clinical and treatment-related factors. Through meta-analysis, the study quantified the relationship between these factors and the risk of infection in patients with multiple myeloma. First, by searching multiple databases, the study ensured the breadth of the literature and the reliability of the data. Second, the study included many studies, involving 4,717 patients, thereby achieving good statistical power. Strict inclusion and exclusion criteria were applied to ensure the quality of the selected literature, providing a solid foundation for the results. Additionally, common risk assessment tools (such as the NOS scoring method) were used to evaluate study quality, and all included studies were high-quality observational studies, further enhancing the credibility of the conclusions.

However, this study also has certain limitations. First, all included studies were observational studies, including cohort studies and case-control studies, which may introduce selection bias and confounding factors. Although we controlled for some confounding factors in the data analysis, due to the limitations of these study designs, potential biases cannot be completely ruled out. Second, differences in infection definitions and classifications across studies may affect the consistency and comparability of results. Although we endeavored to control for these variables, they may still have some impact on the final conclusions. Third, certain key risk factors, such as immunoglobulin levels and vaccination status of patients, were not included in this analysis, which may have prevented us from fully capturing all potential influencing factors. Therefore, future studies should further explore these unconsidered factors and attempt to adopt prospective cohort designs to reduce the interference of confounding factors. Finally, although we assessed publication bias using funnel plots and Egger tests and did not find significant bias, we cannot completely rule out the possibility that some negative results were not published.

## Conclusion

This systematic review and meta-analysis identified several factors associated with increased infection risk in patients with MM, including age >65 years, male sex, smoking, ISS stage III, diabetes, use of immunosuppressive agents, hemoglobin <10 g/dL, and creatinine ≥2 mg/dL. Beyond risk identification, these findings may help contextualize supportive care planning. For instance, patients presenting with multiple risk factors could warrant closer clinical monitoring and more attentive infection surveillance during treatment. The results may also inform risk stratification frameworks that integrate baseline characteristics and treatment exposures when considering the intensity of supportive care.

In addition, although this study does not provide direct evidence for specific interventions, the identified associations may support the rationale for evaluating preventive measures — such as vaccination optimization, monitoring for treatment-related cytopenia, or consideration of guideline-based antimicrobial prophylaxis — within appropriately selected high-risk populations. These implications should be interpreted cautiously given the observational nature and heterogeneity of the included studies.

Future research based on large-scale prospective cohorts is required to validate these associations, develop standardized prediction models, and further clarify the biological mechanisms underlying infection susceptibility in MM.

## Data Availability

The original contributions presented in the study are included in the article/**Supplementary Material**. Further inquiries can be directed to the corresponding author.
