# Supplementary material for: Risk factors for infections in patients with multiple myeloma: a systematic review and meta-analysis
Source: Front Oncol. 2026 Mar 12;16:1726340. doi: 10.3389/fonc.2026.1726340 (PMC13017288; doi:10.3389/fonc.2026.1726340)
Supplement: Supplementary file 1 [file DataSheet1.docx]

Table s1 Search strategy

((("Multiple Myeloma"[Mesh]) OR (((((((((((((((((((((Multiple Myeloma[Title/Abstract]) OR (Multiple Myelomas[Title/Abstract])) OR (Myelomas, Multiple[Title/Abstract])) OR (Myeloma, Plasma-Cell[Title/Abstract])) OR (Myeloma, Plasma Cell[Title/Abstract])) OR (Myelomas, Plasma-Cell[Title/Abstract])) OR (Plasma-Cell Myeloma[Title/Abstract])) OR (Plasma-Cell Myelomas[Title/Abstract])) OR (Myeloma-Multiple[Title/Abstract])) OR (Myeloma Multiple[Title/Abstract])) OR (Myeloma-Multiples[Title/Abstract])) OR (Myeloma, Multiple[Title/Abstract])) OR (Plasma Cell Myeloma[Title/Abstract])) OR (Cell Myeloma, Plasma[Title/Abstract])) OR (Cell Myelomas, Plasma[Title/Abstract])) OR (Myelomas, Plasma Cell[Title/Abstract])) OR (Plasma Cell Myelomas[Title/Abstract])) OR (Kahler Disease[Title/Abstract])) OR (Disease, Kahler[Title/Abstract])) OR (Myelomatosis[Title/Abstract])) OR (Myelomatoses[Title/Abstract]))) AND (("Infections"[Mesh]) OR ((((Infections[Title/Abstract]) OR (Infection[Title/Abstract])) OR (Infestation[Title/Abstract])) OR (Infestations[Title/Abstract])))) AND (("Risk Factors"[Mesh]) OR (((((((((((((((((((Risk Factors[Title/Abstract]) OR (Factor, Risk[Title/Abstract])) OR (Risk Factor[Title/Abstract])) OR (Population at Risk[Title/Abstract])) OR (Populations at Risk[Title/Abstract])) OR (Risk Scores[Title/Abstract])) OR (Risk Score[Title/Abstract])) OR (Score, Risk[Title/Abstract])) OR (Risk Factor Scores[Title/Abstract])) OR (Risk Factor Score[Title/Abstract])) OR (Score, Risk Factor[Title/Abstract])) OR (Health Correlates[Title/Abstract])) OR (Correlates, Health[Title/Abstract])) OR (Social Risk Factors[Title/Abstract])) OR (Factor, Social Risk[Title/Abstract])) OR (Factors, Social Risk[Title/Abstract])) OR (Risk Factor, Social[Title/Abstract])) OR (Risk Factors, Social[Title/Abstract])) OR (Social Risk Factor[Title/Abstract])))


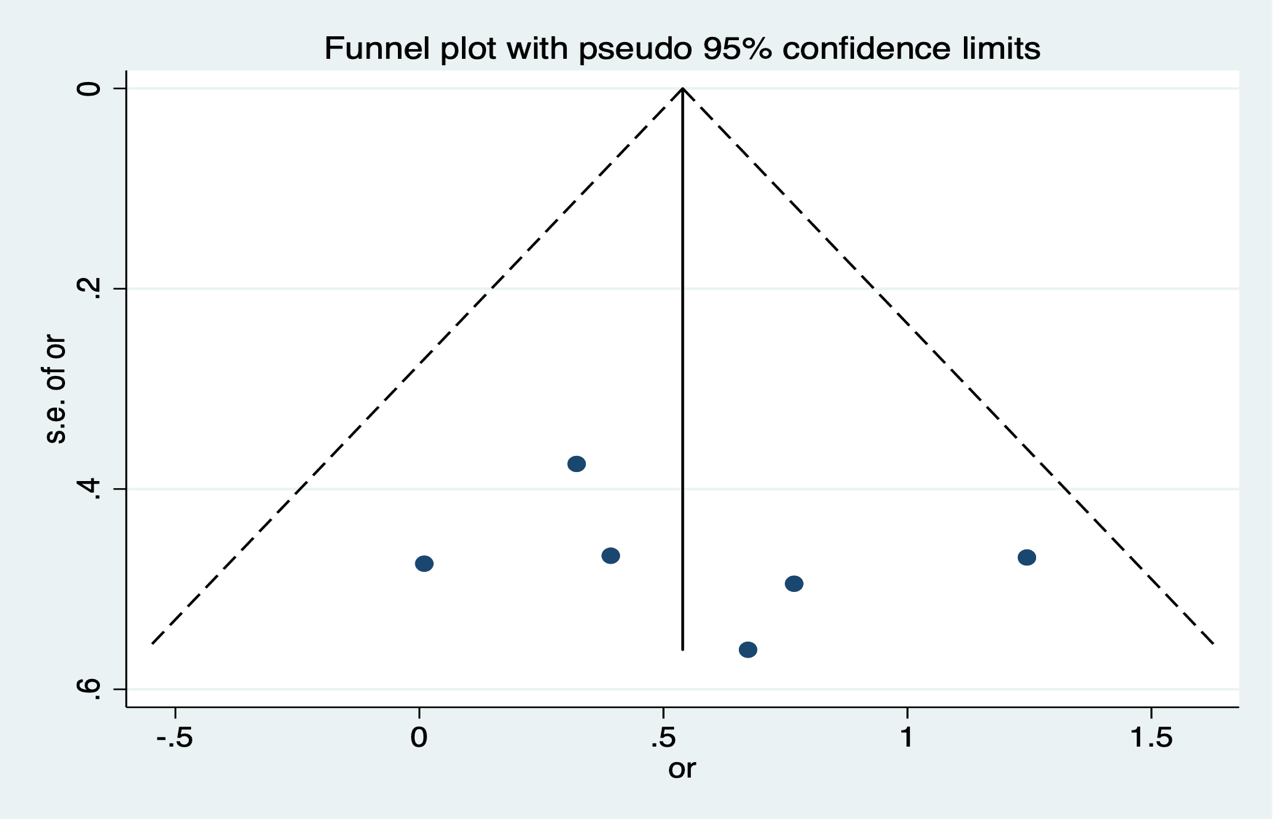


Figure S1 Funnel plot of the meta-analysis of age>65


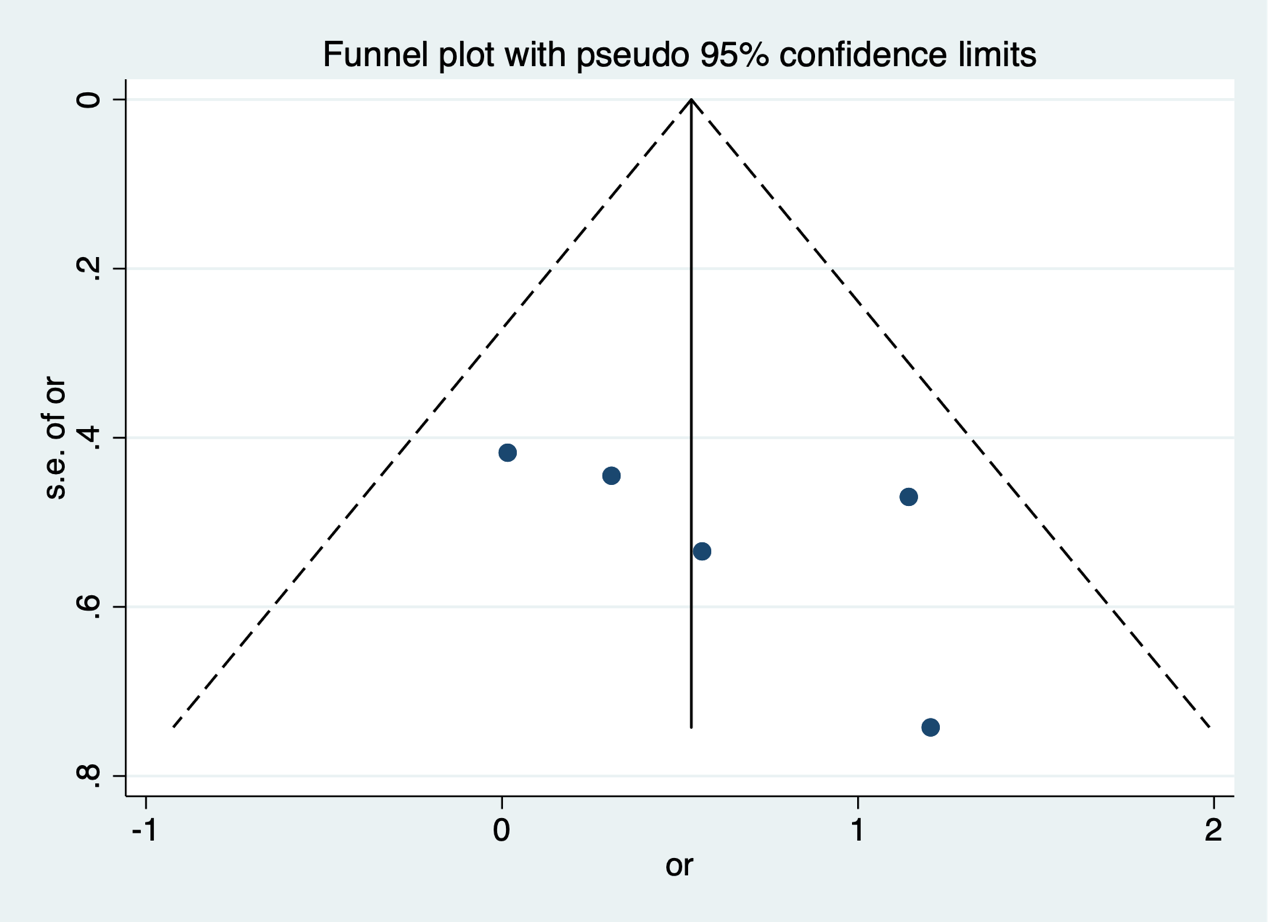


Figure S2 Funnel plot of the meta-analysis of male


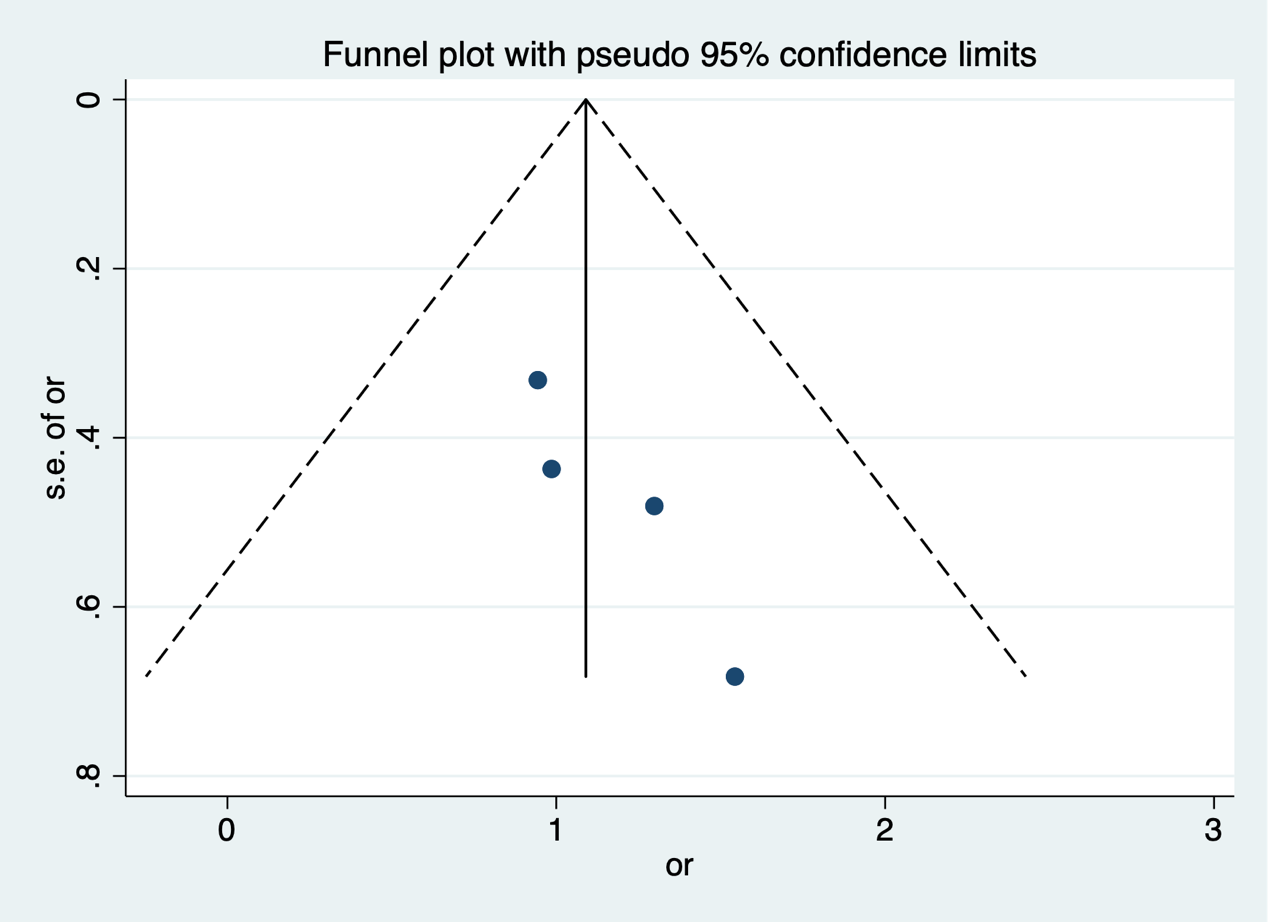


Figure S3 Funnel plot of the meta-analysis of smoking


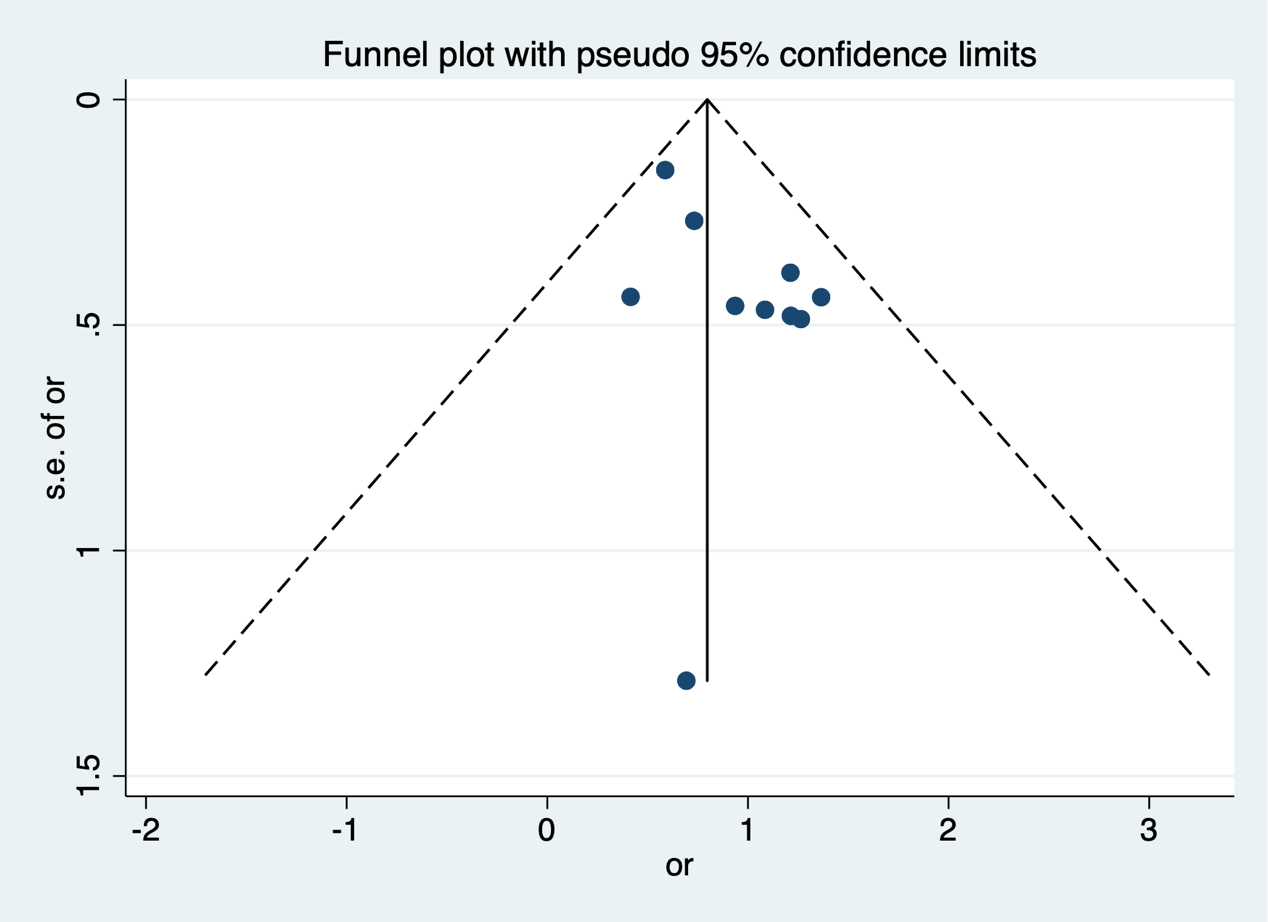


Figure S4 Funnel plot of the meta-analysis of International Staging System III


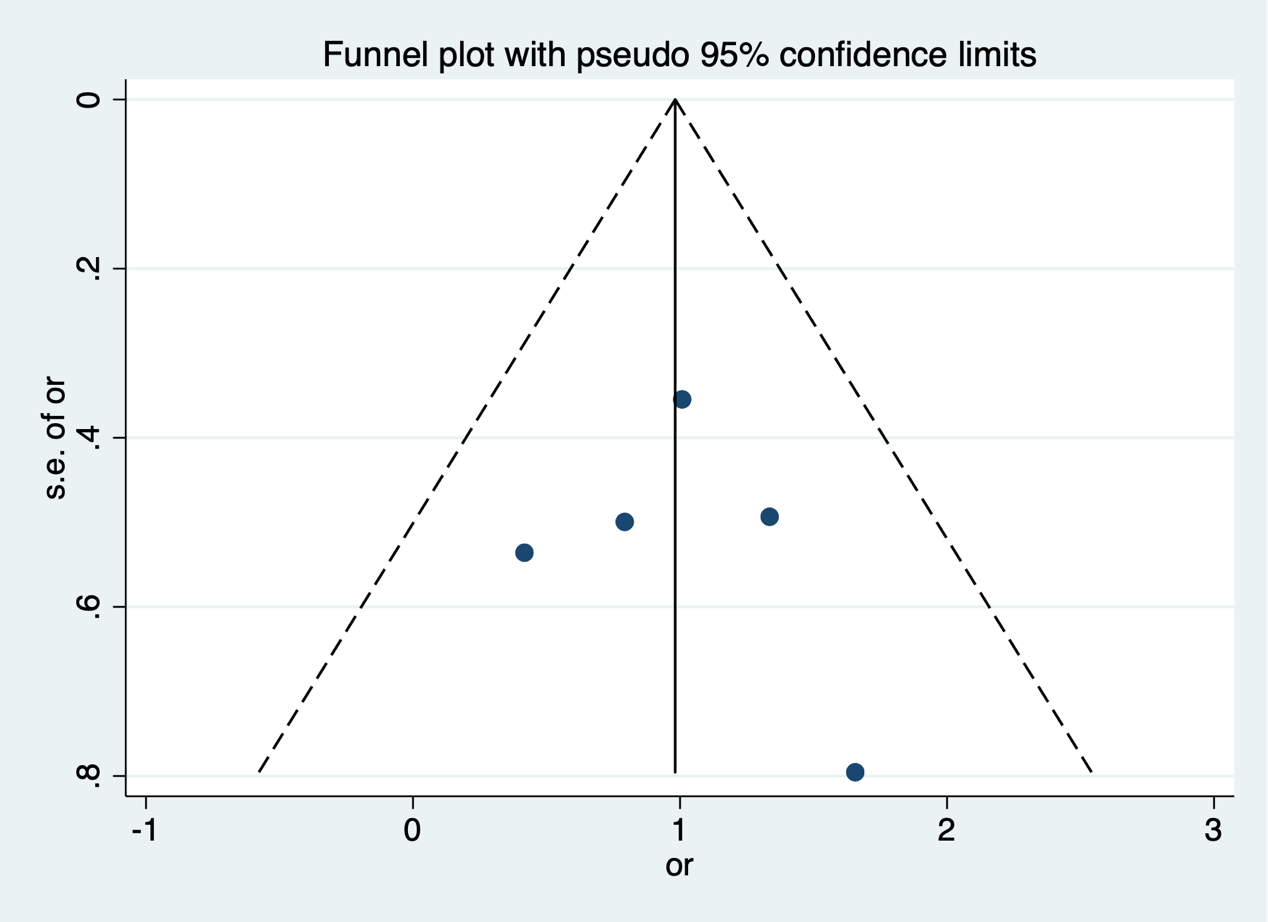


Figure S5 Funnel plot of the meta-analysis of diabetes


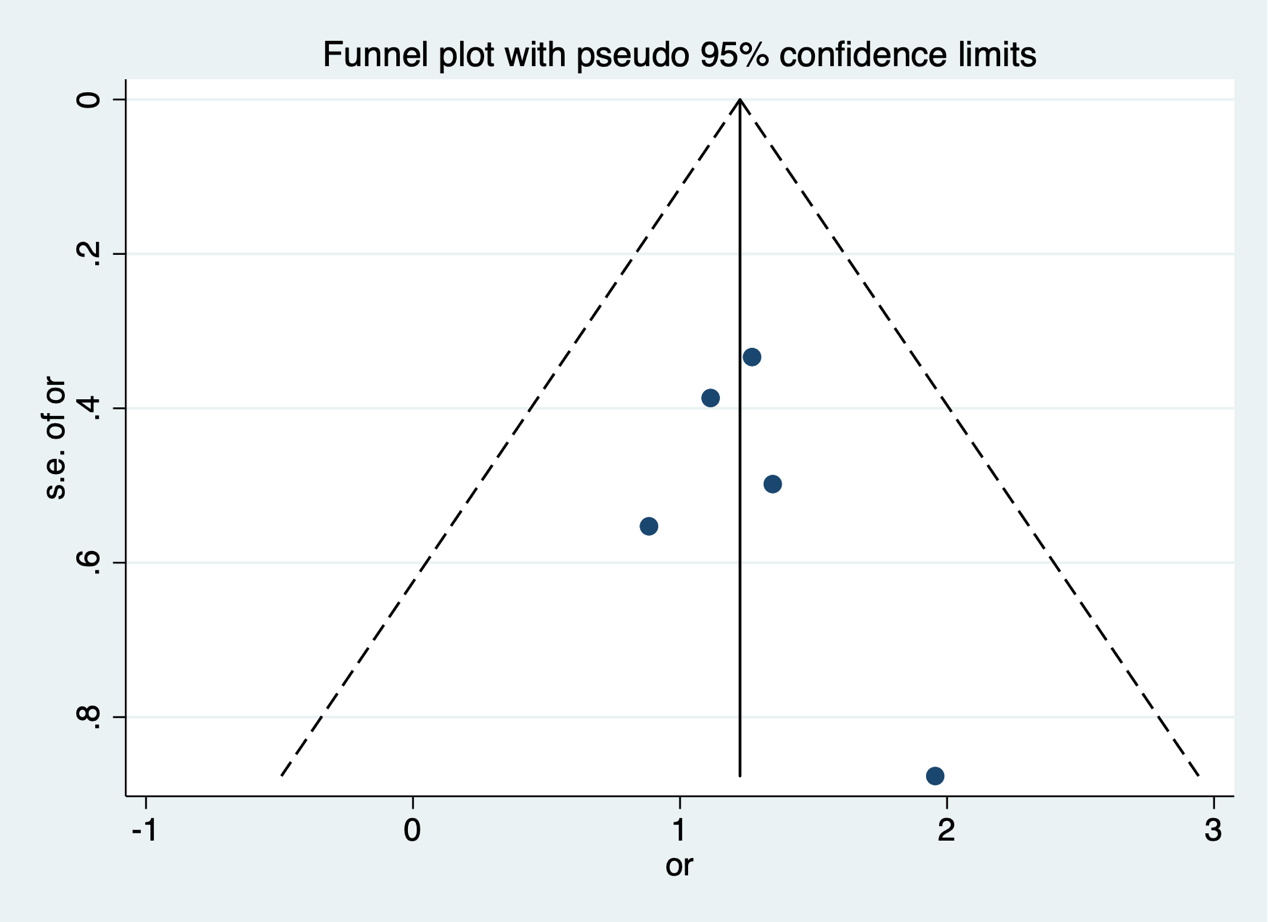


Figure S6 Funnel plot of the meta-analysis of immunomodulatory drugs


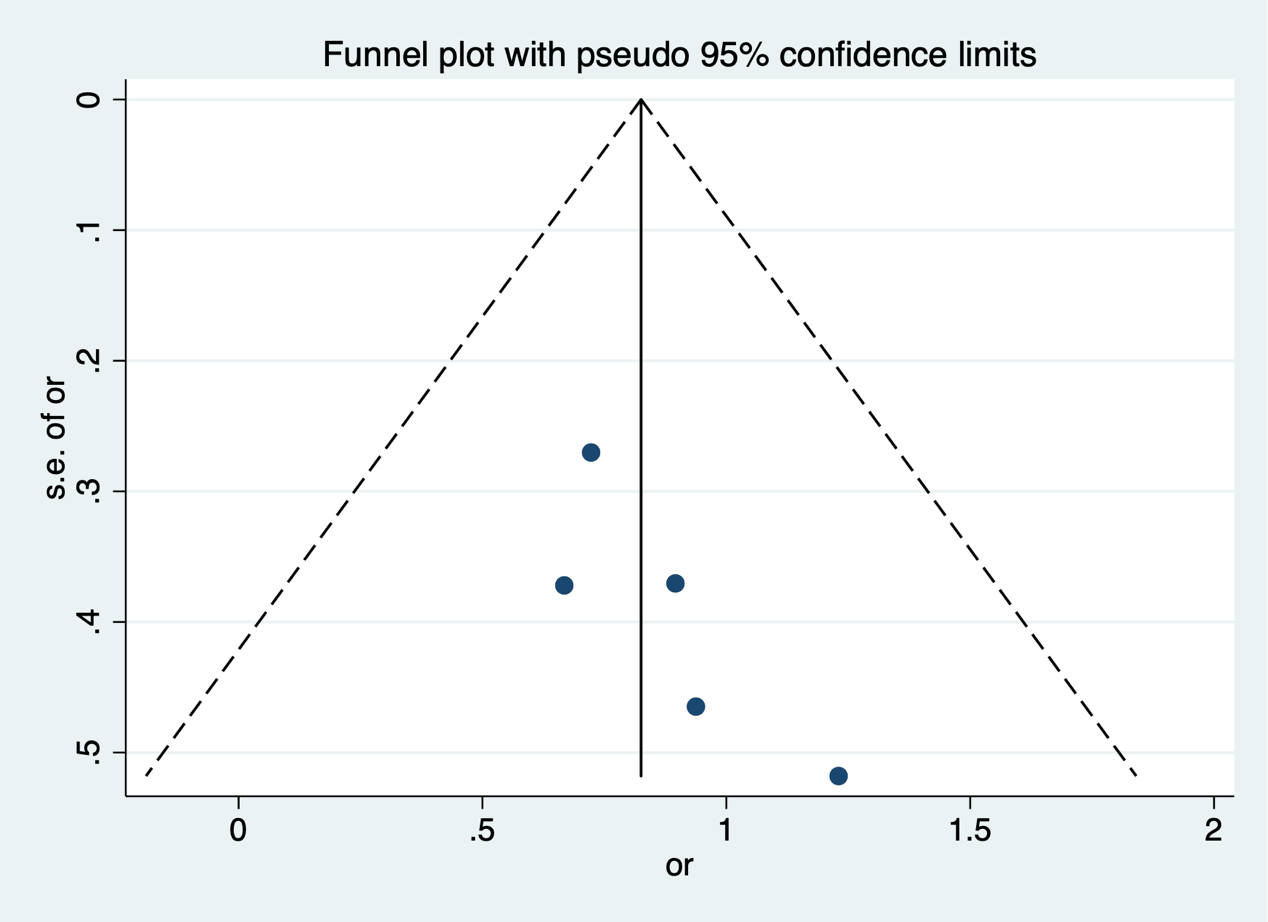


Figure S7 Funnel plot of the meta-analysis of hemoglobin <10g/dL


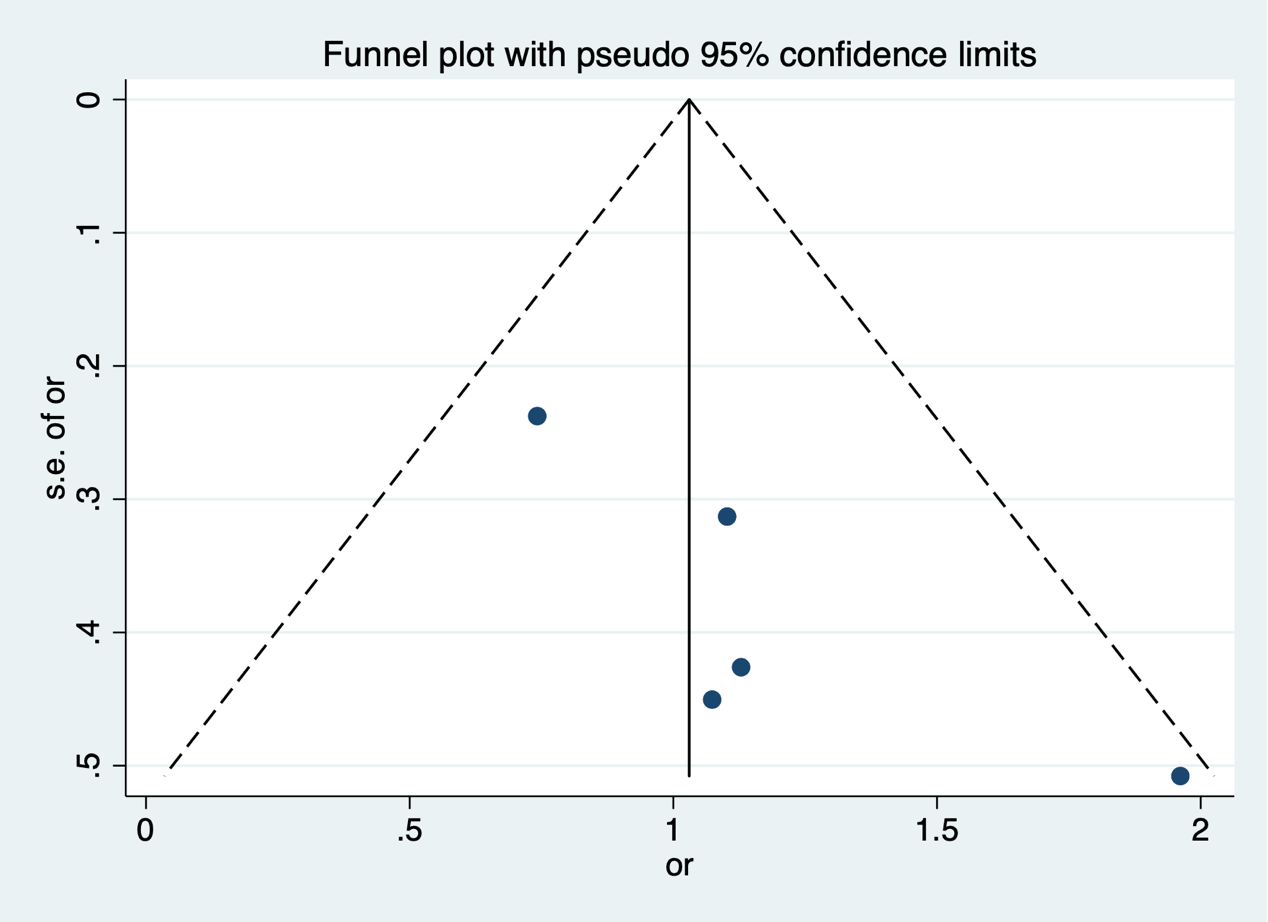


Figure S8 Funnel plot of the meta-analysis of creatinine ≥ 2 mg/dL
